# Comparison of Xpert MTB/RIF Ultra Results of Stool and Sputum in Children with Presumptive Tuberculosis in Southern Ethiopia

**DOI:** 10.3390/tropicalmed8070350

**Published:** 2023-06-30

**Authors:** Yohannes Babo, Bihil Seremolo, Mamush Bogale, Ahmed Bedru, Yasin Wabe, Haba Churako, Alemu Bilat, Tamiru Degaga, Petra de Haas, Edine Tiemersma, Degu Jerene

**Affiliations:** 1 KNCV Tuberculosis Foundation, P.O. Box 703 Code 1110, Addis Ababa, Ethiopia; 2 Worabe Comprehensive Specialized Hospital, Worabe, Ethiopia; 3 Wolaita Sodo University Hospital, Wolaita, Ethiopia; 4 Nigist Eleni Memorial Comprehensive Specialized Hospital, Hossana, Ethiopia; 5 College of Medicine & Health Sciences, Arba Minch University, Arbaminch, Ethiopia; 6 KNCV Tuberculosis Foundation, Maanweg 174, 2516 AB The Hague, The Netherlands

**Keywords:** simple one-step (SOS), stool, Xpert MTB/RIF (Ultra) assay, children

## Abstract

The introduction of stool as a readily obtainable sample and the recently developed simple one-step (SOS) stool processing method on Xpert MTB/RIF Ultra (Xpert Ultra) offer an opportunity for TB diagnosis in children. We conducted this study in secondary health facilities in Ethiopia, which are the first-level referral facilities for childhood TB diagnosis and treatment, with the aim to determine if stool-based TB diagnosis can be performed with a reasonable level of concordance with sputum tests using Xpert MTB/RIF Ultra. Eligible children 0–14 years old with presumptive pulmonary TB were asked to provide stools in addition to routinely requested sputum samples. We determined the level of agreement between the stool and sputum test results. Of the 373 children included in the study, 61% were <5 years of age and 56% were male. Thirty-six children (9.7%) were diagnosed with TB, and all started treatment. The rate of concordance between stool and sputum was high, with a kappa value of 0.83 (*p* < 0.001). There were more Xpert Ultra positive results on stool (n = 27 (7.2%)) than on sputum/NGA (n = 23 (6.2%)). Laboratories in secondary hospitals can perform stool-based TB diagnosis in children, with high concordance between stool and sputum test results reaffirming the applicability of the SOS stool method.

## 1. Introduction

The world is lagging in its commitment to achieving the 2018–2022 global TB target set at the UN high-level meeting (UNHLM) in 2018. This may in part be due to the decline in TB notifications and the increase in TB deaths due to the interruption of TB services caused by the COVID-19 pandemic; only 1.4 million children were treated for TB between 2018 and 2020, which is only 41% of the five-year target of 3.5 million [1]. The number of missed TB patients is disproportionally high among young children [2]. The diagnosis of TB is more challenging, especially in young children, because they often do not produce adequate sputum and often have non-specific symptoms and signs. Furthermore, the diagnosis of TB in children is hindered by the low sensitivity and limited accessibility of microbiological testing and the low probability of obtaining a specimen. Most children with presumptive TB seek care at primary health facilities and face diagnostic delays of more than a month, during which, multiple providers are met, and the patients undergo several diagnostic tests, most of them being inappropriate for TB diagnosis [3]. Alternative specimens, such as gastric aspirates or induced sputum, require specific skills and equipment which are usually not available in lower-level health care facilities and in remote settings.

Ethiopia developed its national childhood TB roadmap in 2015 and last updated it in 2019. The key strategies outlined in the roadmap are integrating TB screening, diagnosis, and prevention services into IMNCI (Integrated Management of Newborn and Childhood Illnesses) clinics at the primary health care level and intensifying contact investigations at the TB clinics [4]. These strategies supported the integration of childhood TB services into other childcare services addressing children’s particular needs. Key achievements were increased national leadership in addressing childhood TB and the development of working groups focusing on childhood TB, increasing recognition of the importance of integrating childhood TB into IMNCI clinics, and the revision of the IMNCI register to include TB screening. A study carried out in Addis Ababa, Ethiopia, to evaluate TB screening integration into IMNCI demonstrated the feasibility of the strategy and resulted in improved TB screening, the identification of presumed TB cases, and TB case detection [5].

Despite concerted efforts made to address childhood TB, the lack of child-friendly diagnostic samples other than sputum to confirm TB diagnosis and perform drug susceptibility tests remains a key barrier in operationalizing the strategies in the national roadmap. Since 2020, stool Xpert MTB/RIF (Xpert) (Cepheid, Sunnyvale, CA, USA) has been recommended by the world health organization (WHO) as an alternative sample to diagnose TB in children [6]. Since 2022, stool has also been recommended to be tested using the more sensitive Xpert MTB/RIF Ultra (Xpert Ultra) [7,8]. A simple stool processing method called the simple one-step (SOS) stool method is gaining popularity because of its ease of implementation at peripheral sites [9,10]. The method is as simple as sputum processing, requires no additional equipment or supplies, and can be performed at all sites where an Xpert instrument is functional [6,10,11]. The method was shown to have similar sensitivity and specificity compared to other stool processing methods [7,9,12].

While we earlier confirmed the utility of the SOS stool method in different settings [13,14,15], further evidence of its operational feasibility in lower-level health facilities is needed. In this study, we determined if the SOS stool method can be implemented in secondary-level health facilities under routine settings and the (incremental) yield of using stool as an alternative sample in children. The findings of this study will provide additional evidence on the scalability of stool-based testing using the Xpert Ultra cartridge to address the diagnostic difficulty in childhood TB care in a high-TB-burden and resource-limited setting.

## 2. Materials and Methods

### 2.1. Study Design and Setting

A cross-sectional study was conducted in four selected hospitals in the Southern Nations, Nationalities, People’s Region (SNNPR), Ethiopia, from October 2021 to December 2021. The study recruited children seeking care in the outpatient clinic and identified in the selected health facilities as presumptive TB patients as per the national algorithm. The four hospitals were selected because of a relatively high TB notification rate among children, serving a catchment population with low socioeconomic status and experience of research participation in addition to the availability of a GeneXpert instrument on-site. These hospitals are the first-level referral sites for the diagnostic evaluation and treatment of TB in children referred from primary health facilities.

### 2.2. Sample Size and Sampling

Assuming a positive discordance rate of 10%, a negative discordance rate of 5%, and a study power of 90%, at α = 5%, the calculated sample size for the current study was 627 children with presumptive TB [16]. Children with presumptive pulmonary TB visiting the selected hospitals during the study period whose parents consented for participation were recruited consecutively. Since this sample size was not achieved due to the lower enrollment rate than anticipated, we performed power analysis with the sample size achieved, which yielded 70% power. 

### 2.3. Study Population

We consecutively enrolled children up to 14 years old who visited the selected health care facilities and were identified as presumptive pulmonary TB patients (clinical symptoms suggestive of pulmonary TB (PTB), (i.e., cough for more than two weeks; weight loss/failure to thrive (FTT); reduced playfulness; fever/night sweats) and/or contact history with a registered TB case and/or chest X-ray (CXR) suggestive of TB) by the clinician. Critically ill patients (including those in a coma, terminally ill due to chronic debilitating co-morbidities, or other conditions determined to be “critical” by the treating physician), and those whose parents/caregivers did not provide informed consent were excluded from the study.

### 2.4. Study Procedures

#### Participant Enrollment

The parents/caregivers of eligible children were informed about the study by the data collectors from the health facility during their medical visit. They were provided with an information sheet containing the details of the study. Written parental consent was taken if they agreed to their child’s participation in the study. In addition to routinely requested sputum samples (spontaneous/naso gastric aspirate (NGA)) and other tests (CXR if available), all children were requested to submit one stool sample. Parents/caregivers were instructed by the laboratory personnel on how to collect sputum and stool samples on site and were provided with stool containers to allow the collection of stool samples for same-day testing.

### 2.5. Laboratory Procedures

Dedicated laboratory personnel received the sputum and stool samples and recorded the demographic data in the Xpert MTB/RIF register. National standard operating procedures were used to process and test the sputum using an Xpert-Ultra assay, which follows the procedure as provided by the manufacturer. Briefly, sample reagent (SR) was added in a 2:1 ratio to the sputum specimen. It was mixed well twice during the 15 min incubation time at room temperature. Two milliliters (ml) of the mixture was transferred into a test cartridge. The test cartridge was loaded into the GeneXpert instrument. *M. tuberculosis* (MTB) positivity and its rifampicin (RIF) resistance were determined within 2 h. For stool, the SOS stool processing method was followed [17]. In short, 0.8 g of solid or 2mL of liquid stool was added to the SR bottle which comes with every Xpert-Ultra cartridge, and the mixture was then vigorously shaken by hand for 30 s, then incubated for 10 min, then shaken again by hand for 30 s, and incubated for another 10 min to allow debris to settle. Next, 2 mL of the supernatant was transferred to the Xpert-Ultra cartridge and analyzed using the GeneXpert instrument. The semi-quantitative levels of Xpert-Ultra results were trace, very low, low, medium, or high. The non-determinate Xpert-Ultra results (invalid, no result, and errors) were repeated once to define the final test outcome. The diagnosis of TB was made when MTB was detected from sputum samples or based on the clinical assessment of the clinician according to the national guidelines.

### 2.6. Data Management and Statistical Analysis

Detailed clinical and demographic information about the patients and Xpert-Ultra test results of their samples were collected using structured paper forms developed for the study. The paper-based records were then entered using a KoBo Toolbox [18] data entry sheet developed for this study. The quality of data entry was validated by cross-checking selected key variables in the database with the information on the paper forms for 10% of the participants. The data were finally analyzed using SPSS (version 20). Descriptive analyses were used for participant characteristics. We further used Cohen’s kappa statistic to measure the level of agreement between sputum and stool Xpert-Ultra testing.

### 2.7. Ethical Considerations

The study protocol was approved by the SNNPR Ethical Review Committee (የወ6-19/1826). Participants’ information was kept confidential. We also did not include any names or other personal identifying information of the participant in the digital files. The informed consent and paper forms with personal identifying information were stored in a lockable cabinet.

## 3. Results

Of 373 children with presumptive TB included in the study, 61% were <5 year of age and 56% were male. The median age was 3 (IQR: 1–8). Most patients reported chronic cough (98%), followed by fever (88%) and weight loss or failure to thrive (69%), and 21% had contact history with a known TB patient. HIV status was available for 239 (64%), of whom, 4 (1.7%) were HIV-infected (Table 1).

In total, 36 children (9.6% of 373) were diagnosed with TB, of whom, 23 (64%) had bacteriologically confirmed TB (positive Xpert-Ultra sputum). A valid test result was obtained for 368/373 children both for stool and the sputum/NGA sample with a concordant result for 360 (96.5%, 95% CI 71–98%) children. The rate of concordance between stool and sputum was high, with a kappa value of 0.83 (Table 2). Discordant results were found in six children with Xpert-Ultra stool positivity (three: MTB detected, trace; two: MTB detected, low; and one: MTB detected, high) and negative Xpert-Ultra sputum results. A discordant result was also found in two children having negative Xpert-Ultra stool and positive Xpert-Ultra sputum results (both MTB detected, trace).

Of the six children with MTB detected in stool only, four were children under the age of five. Two children had a TB contact history and several symptoms suggesting TB, while the other half only had several symptoms suggesting TB. Out of the six children, only one child was clinically diagnosed with TB and started treatment during the data collection period. The demographic and clinical characteristics of children with MTB detected in stool specimens are depicted in Appendix A. Rifampicin resistance was not detected in the sputum or stool samples. Out of the 23 children with MTB detected in sputum, 1 (4.3%) had indeterminate rifampicin resistance. MTB was not detected in the stool of this child. Meanwhile, out of the 27 children with MTB detected in stool, 4 (14.8%) had indeterminate rifampicin resistance. Three of the indeterminate results had “trace” and one had “low” semi-quantitative values in their stool test. Four children started treatment for drug-susceptible TB; for three, this was based on their sputum result, and one had clinically diagnosed TB. Repeated tests from a fresh sputum sample did not detect rifampicin resistance for the three children. Repeat tests were not carried out in the remaining two children. The demographic, clinical, and bacteriological characteristics of children with indeterminate Rif resistance are depicted in Appendix B.

MTB was detected in both the sputum and stool of 1/4 (25%) of HIV-infected children (very low on sputum and trace on stool). This child was a one-year-old with cough, fever, and weight loss; had contact history with pulmonary TB patients; and was started on treatment. In the HIV-uninfected children, stool testing detected MTB in 23/235 (9.8%), while sputum testing detected MTB in 21/235 (8.9%). Among children under the age of 5 years, stool testing detected MTB in 14/27 (51.9%) versus MTB detection in 12/23 (52.2%) in the sputum testing (Table 3).

Of the 371 Xpert-Ultra tests conducted on sputum/NGA samples, 1 (0.27%) resulted in non-determinate test results (invalid). Of the 373 Xpert-Ultra tests on stool samples, 9 (2.4%) resulted in non-determinate results (7: errors; 1: invalid; and 1: no result). The error codes for non-determinate stool Xpert-Ultra results were 5011, 2037, 2097, 2005, 2008, or 5007 at initial testing. The difference in the proportions of non-determinate test results for the Xpert-Ultra testing of sputum/NGA samples versus the testing of stool samples with the SOS stool method was statistically significant (*p* = 0.048). For six (75%) out of eight stool samples in which the Xpert-Ultra test was repeated (for the sample with ‘no result’, the test was not repeated), the repeat test led to valid results.

Overall, there were more positive Xpert-Ultra MTB test results on stool (n = 27 (7.2%) of all results) than on sputum/NGA (n = 23 (6.2%) of all results, *p* < 0.001). The semi-quantitative result of the Xpert-Ultra of the stool and the sputum/NGA specimen had no significant difference. The Xpert-Ultra trace rate on stool was 6/27 (22.2%). Six children with MTB trace calls detected in the stool sample were under the age of five years. Out of the six children with trace calls in the stool, four had very low MTB detected via Xpert-Ultra in the sputum and two had no MTB detected. The two children had TB-suggestive symptoms and one had TB contact history, but they were not clinically diagnosed to have TB (Table 4).

## 4. Discussion

This study assessed the rate of agreement between sputum/NGA and stool sample results on Xpert-Ultra in four hospitals in southern Ethiopia. Our study has some key findings. First, the Xpert-Ultra results on stool samples had very good concordance with the sputum/NGA sample results. Second, there was a low rate of non-determinate results for stool specimens related to stool processing, even though this was slightly higher than that for sputum specimens. Third, MTB was more often detected in the stool samples than in the sputum samples. Fourth, the semi-quantitative Xpert Ultra results of sputum/NGA samples were not different from those measured via the Xpert Ultra testing of stool samples.

The very high concordance rate between Xpert-Ultra results on stool and sputum/NGA samples implies that stool samples yield a comparable result with that of sputum/NGA samples when carried out in a routine setting. A study carried out in China, a high-TB-burden country, reported the moderate and good quality of the agreement between stool and sputum specimens in Xpert-Ultra assays [19]. The stool processing methods used in the Chinese studies were different to the one we report in the current study. In the Chinese study, stool samples were homogenized and suspended in a phosphate buffer and glass beads and vortexed and processed using a filter screen. Another small study from a tertiary hospital in Indonesia with a two-step stool processing method showed 89% (95% CI 71–98%) concordant results between stool and respiratory samples [20]. A study from Pakistan reported the comparable performance and good agreement between the Xpert testing of stool and sputum/NGA in a cohort of children with a high probability of pulmonary TB, though the stool processing method was different, including the stool volume used, i.e., 0.15 g of stool [21].

The rate of non-determinate stool Xpert Ultra results was within the acceptable range, and repeated testing of initial non-determinate stool results led to a valid result in almost all cases. There is a need to closely monitor the rate of non-determinate test results of the stool Xpert testing, as is carried out for sputum Xpert testing, and take corrective actions. The on-site training and supervision of laboratory staff is critical to improve performance and reduce non-determinate results overtime. One reason for invalid/error results in stool-based testing is the presence of PCR inhibitors that are naturally more present in stool compared to sputum; another reason may be that when not carefully transferring the supernatant and when the sedimentation process in the SOS stool method is disturbed, debris of the stool might travel into the cartridge, which leads to clotting of the micro fluid filter in the Xpert-Ultra cartridge, leading to specific errors like error 2008 and 5006/5007. It is therefore important to distinguish the errors that occur due to stool processing and error codes that are related to the GeneXpert instrument or cartridge failure. A study by de Haas et al. showed a non-significant difference in the stool processing non-determinate results of the Xpert testing of NGA versus stool samples, while the rate of non-determinate results decreased over time, probably due to experience with the SOS stool method among lab technicians having increased in Vietnam [9]. A study in Southwest Ethiopia in a teaching and referral hospital using the SOS stool method documented a 4.6% (7/152) rate of error/invalid results with the Xpert assay [22]. This difference could be because Xpert-Ultra is less likely to produce invalid or error results than Xpert, offering better performance and making it a better choice for the diagnosis of tuberculosis. Another study in China found a higher frequency of invalid test results from stool samples than from GA. The processing method used in this study, which involved using phosphate buffer for stool processing, may have inhibited the PCR reactions [19].

In this study, stool specimens yielded a higher proportion of MTB detected than sputum specimens. This could be since stool samples may contain MTB DNA from sites other than the lungs, such as the abdominal lymph nodes, intestines, and pleura. Moreover, this can be explained by the low quantity and/or poor quality of the sputum/NGA samples taken from children in the younger age group. A similar finding was reported by de Haas et al. in Ethiopia and Vietnam, where children had MTB detected in their stool but not in their sputum/NGA sample [9,11]. There was no notable difference in the performance of stool testing as compared to sputum testing by the age, TB contact history, presenting symptoms, and HIV status of children (the number of HIV-infected children was too low to make a comparison in this study). Though not statistically significant, studies from South Africa [23] and Zimbabwe [24] reported that Xpert MTB/RIF on stool was more sensitive in HIV-infected than in HIV-uninfected children. There is very limited information (published) on Xpert stool testing for extrapulmonary TB. The recommendations made so far only focus on the use of stool-based testing for pulmonary TB. In a study carried out in Pakistan among adults (>18 years of age) with suspected intestinal tuberculosis on clinical grounds, Xpert stool results were positive in 20% of the patients (n = 100). There is a possibility that positive Xpert stool results could be due to swallowed sputum; however, in this study, out of 4 patients who had positive sputum Xpert, only 1 had a positive stool Xpert result, while 17 of the patients positive stool Xpert results had negative Xpert results in sputum [25].

Another important finding in our study was that the semi-quantitative Xpert-Ultra results were not different between the stool and sputum samples. Out of the six trace calls in the stool, four had very low MTB in the sputum and two had no MTB detected. The two children had multiple TB-suggestive symptoms and were not clinically diagnosed to have TB. A study in Bangladesh reported that the proportion of trace calls was higher in stool specimens compared to induced sputum [21], while a study by Sun in China found that the semi-quantitative scale used for Xpert-Ultra on stool samples correlated with that of Xpert-Ultra using gastric aspirate [19]. Detecting MTB from swallowed sputum in the stool in the lower scale of the semi-quantitative scale (low, very low, and trace call) could be due to the paucibacillary nature of TB in children. In addition, MTB is more diluted in intestines as sputum gets mixed with food, PCR is inhibited due to substances in stool, and it is less easy to “free” the MTB bacilli from stool than from sputum.

In this study, we observed a high proportion of indeterminate rifampicin results in stools, in line with the high trace semi-quantitative results. When the Xpert result is MTB—trace, the Rif results are always indeterminate. The reason is that with a trace, the RpoB gene is not detected, so there is no way the test can give any interpretation on the rifampicin resistance result. This is generally caused by a paucibacillary TB load in the sample due to insufficient signal detection [26]. Therefore, alternative specimens may need to be collected in persons with a high likelihood of drug-resistant TB. Walters reported 33.3% Xpert MTB/RIF indeterminate results in stool samples [27]. From a systematic review of Xpert MTB/RIF and Xpert MTB/RIF Ultra assays for active tuberculosis and rifampicin resistance in children, few Xpert MTB/RIF results were indeterminate for the detection of rifampicin resistance [28]. Vibol reported 14% Xpert MTB/RIF Ultra indeterminate results from individual testing and none for Xpert MTB/RIF [29].

One limitation of our study was that we have no follow-up data for those children with MTB detected in stool reporting clinical symptoms of TB. Thus, we do not know if they were diagnosed at a later point in time. This was because stool was not yet recommended as an initial sample for testing in Ethiopia at the time of the study. This study on the SOS stool processing method was successfully implemented in peripheral laboratories under the routine conditions of the resource-limited setting. A second limitation was we had a sample size less than anticipated as we were not able to achieve the planned sample size of 627 due to unforeseen external factors. However, this has minimal consequences on the interpretation of the concordance rate, as the discordant rate assumptions used to calculate the sample size were much higher than what we observed in this study.

## 5. Conclusions

The SOS stool method with Xpert-Ultra for TB detection yields a comparable result with sputum Xpert-Ultra testing. Stool can be used as an alternative to respiratory specimens and was shown to be a non-invasive test for patients who cannot provide sputum. Hence, the SOS stool method is applicable to expand childhood TB services at secondary health care levels. Further studies on the applicability of stool-based testing for TB diagnosis among HIV-infected children are warranted, as these children benefit most from this non-invasive and innovative method.

## Figures and Tables

**Table 1:** Baseline characteristics of the study participants (n = 373).

Characteristic		Number (%)
Age (in years)	<5	226 (60.6)
	5–14	147 (39.4)
	Median (IQR)	3 (1–8)
Sex	Female	163 (43.7)
	Male	210 (56.3)
Presenting symptoms	Chronic cough	365 (97.8)
	Fever	327 (87.7)
	Weight loss or failure to thrive	258 (69.2)
	Night sweats	205 (55.0)
	Contact history	78 (20.9)
HIV status	Negative	235 (63.0)
	Not determined	134 (35.9)
	Positive	4 (1.1)

**Table 2:** Comparison of Xpert-Ultra results of stool and sputum/NGA from children with presumptive TB.

MTB Detected from Sputum/NGA	MTB Detected from Stool		
	No	Yes	Total
No	339	6	345
Yes	2	21	23
Total	341	27	368
Kappa value: 0.83; 95% CI 0.71–0.94			

**Table 3:** Characteristics of children diagnosed with TB via sputum and stool samples.

Characteristics	No of Children with MTB Detected in Sputum (n = 23)	No of Children with MTB Detected in Stool (n = 27)	*p*-Value
Age, median (IOR)	3 (1–7)	3 (1–7)	
Sex			
Female	10 (43.5%)	11 (40.7%)	0.85
Age			
<5	12 (52.2%)	14 (51.9%)	0.98
>5–14	11 (47.8%)	13 (48.1%)	
History of TB contact			
Yes	16 (69.6%)	18 (66.7%)	0.83
HIV status			
Positive	1 (4.3%)	1 (3.7%)	
Presenting symptoms			
Cough	23 (100%)	27 (100%)	
Fever	23 (100%)	26 (96.3%)	
Weight loss	23 (100%)	24 (88.9%)	
Night sweats	21 (91.3%)	22 (81.5%)	0.56 *

* Fisher’s exact test.

**Table 4:** Semi-quantitative categories of Xpert-Ultra assays on sputum and stool specimens among bacteriologically confirmed PTB patients in children from 4 hospitals in SNNPR, Ethiopia, October–December 2021.

Sputum Xpert Ultra-Result	Negative	Trace	Stool Xpert MTB/RIF Ultra-Result			
			Very Low	Low	Medium	High
Negative		2	0	3	0	1
Trace	2	0	0	0	0	0
Very low	0	4	3	0	0	0
Low	0	0	2	6	0	0
Medium	0	0	0	2	0	0
High	0	0	1	1	1	1

## Data Availability

The data presented in this study are available on request from the corresponding author. The data are not publicly available due to privacy and ethical restrictions.

## Appendix A. Demographic and Clinical Characteristics of Children with MTB Detected in Stool Only

**Age in Years**	**Sex**	**Cough**	**Fever**	**Weight Loss or FTT**	**Contact History**	**Was TB Diagnosed Clinically?**	**Was TB Treatment Started?**	**Xpert Ultra Result**
2	Male	Yes	Yes	Yes	Yes	Yes	Yes	MTB detected low
9	Female	Yes	Yes	No	Yes	No	No	MTB detected low
0	Male	Yes	No	No	Yes	No	No	MTB detected trace
0	Male	Yes	Yes	Yes	No	No	No	MTB detected low
3	Female	Yes	Yes	Yes	No	No	No	MTB detected trace
13	Male	Yes	Yes	No	No	No	No	MTB detected high

## Appendix B. Demographic, Clinical, and Bacteriological Characteristics of Children with Indeterminate Rif Resistance Result

**Age in Years**	**Sex**	**Cough**	**Fever**	**Weight Loss or FTT**	**Contact History**	**HIV Status**	**Sputum Xpert Result**	**Stool Xpert Result**	**Was TB Treatment Started?**
2	Male	Yes	Yes	Yes	Yes	Neg	Not detected	Low Rif indeterminate	Yes *
2	Male	Yes	Yes	No	Yes	Neg	Very low Rif not detected	Trace Rif indeterminate	Yes
2	Male	Yes	No	No	Yes	Neg	Very low Rif not detected	Trace Rif indeterminate	Yes
0	Male	Yes	No	No	No	-	Not detected	Trace Rif indeterminate	No
2	Male	Yes	Yes	Yes	No	Neg	Trace Rif indeterminate	Not detected	Yes
- HIV test not carried out. * Clinically diagnosed.									
